# Platelet Activation Pathways Controlling Reversible Integrin αIIbβ3 Activation

**DOI:** 10.1055/s-0044-1786987

**Published:** 2024-06-22

**Authors:** Jinmi Zou, Siyu Sun, Ilaria De Simone, Hugo ten Cate, Philip G. de Groot, Bas de Laat, Mark Roest, Johan W.M. Heemskerk, Frauke Swieringa

**Affiliations:** 1 Platelet (patho)physiology, Synapse Research Institute, Maastricht, The Netherlands; 2 Department of Biochemistry and Internal Medicine, Maastricht University Medical Center + , Maastricht, The Netherlands

**Keywords:** integrin αIIbβ3, glycoprotein VI, P-selectin, protease-activated receptors, protein kinase C

## Abstract

**Background** Agonist-induced platelet activation, with the integrin αIIbβ3 conformational change, is required for fibrinogen binding. This is considered reversible under specific conditions, allowing a second phase of platelet aggregation. The signaling pathways that differentiate between a permanent or transient activation state of platelets are poorly elucidated.

**Objective** To explore platelet signaling mechanisms induced by the collagen receptor glycoprotein VI (GPVI) or by protease-activated receptors (PAR) for thrombin that regulate time-dependent αIIbβ3 activation.

**Methods** Platelets were activated with collagen-related peptide (CRP, stimulating GPVI), thrombin receptor-activating peptides, or thrombin (stimulating PAR1 and/or 4). Integrin αIIbβ3 activation and P-selectin expression was assessed by two-color flow cytometry. Signaling pathway inhibitors were applied before or after agonist addition. Reversibility of platelet spreading was studied by microscopy.

**Results** Platelet pretreatment with pharmacological inhibitors decreased GPVI- and PAR-induced integrin αIIbβ3 activation and P-selectin expression in the target order of protein kinase C (PKC) > glycogen synthase kinase 3 > β-arrestin > phosphatidylinositol-3-kinase. Posttreatment revealed secondary αIIbβ3 inactivation (not P-selectin expression), in the same order, but this reversibility was confined to CRP and PAR1 agonist. Combined inhibition of conventional and novel PKC isoforms was most effective for integrin closure. Pre- and posttreatment with ticagrelor, blocking the P2Y _12_ adenosine diphosphate (ADP) receptor, enhanced αIIbβ3 inactivation. Spreading assays showed that PKC or P2Y _12_ inhibition provoked a partial conversion from filopodia to a more discoid platelet shape.

**Conclusion** PKC and autocrine ADP signaling contribute to persistent integrin αIIbβ3 activation in the order of PAR1/GPVI > PAR4 stimulation and hence to stabilized platelet aggregation. These findings are relevant for optimization of effective antiplatelet treatment.

## Introduction

One of the fundamentals of platelet aggregation is the conformation change of the abundantly expressed integrin αIIbβ3 from a resting and closed conformation to an activated and extended conformation. 1 2 This change, induced by essentially all platelet agonists, allows fibrinogen to bind to the activated integrin molecules, which is a sufficient condition for platelet aggregate and thrombus formation. 3 In a recent literature review, 4 evidence was summarized that the agonist-induced activation of αIIbβ3 and hence platelet aggregation are intrinsically reversible processes. Other findings stipulate that autocrine released adenosine diphosphate (ADP) sustains the aggregation process, in particular induced via the protease-activated receptors (PARs) of thrombin. 5 6 7 Reversibility in protein phosphorylation and further signaling is also seen upon platelet aggregation via the collagen receptor glycoprotein VI (GPVI). 8 9 The importance of such reversibility for platelet physiology appeared from the finding that previously activated platelets (with transiently active integrins) can be reactivated to maintain thrombus formation, 10 although still unclear are the precise signaling mechanisms leading to a transient or a continued αIIbβ3 activation.

Antagonism of protein kinase C (PKC) and protein kinase A on the integrin activation mechanism, using stimuli of these serine/threonine protein kinases, was already described in 1991. 11 Later, several PKC isoforms appeared to contribute to the aggregation process, 12 the roles of which were enforced by moderate rises in cytosolic [Ca ^2+^ ] _i_ . 13 14 Accordingly, platelet aggregation is provoked by the general PKC activator, phorbol myristate acetate (PMA), and the intracellular Ca ^2+^ -ATPase inhibitor thapsigargin. The literature data pointed to partly overlapping and partly antagonistic roles of the conventional, Ca ^2+^ -dependent PKC isoforms α/β and the novel, Ca ^2+^ -independent PKC isoforms δ/θ. 15 16 17 Not much is known of other signaling elements and pathways contributing to the platelet aggregation response, 4 with the exception of phosphoinositide 3-kinase (PI3K) activation. 7 18 19

In the present paper, we studied the transiency of integrin activation for platelets stimulated with the GPVI agonist collagen-related peptide (CRP) and the PAR agonists thrombin (PAR1/4), TRAP6 (PAR1), and AYPGKF (PAR4). By applying pharmacological inhibitors either before or after an agonist, we investigated herein the possible roles of PKC and PI3K isoforms and other pathways. As the integrin activation process is essential for thrombosis and hemostasis, the gained insight into these pathways is critical to optimize effective antiplatelet treatment.

## Materials and Methods

### Materials

Thrombin was obtained from Enzyme Research Laboratories (South Bend, Indiana, United States). The platelet agonist thrombin receptor-activating peptide 6, TRAP6 (SFLLRN, PAR1), was purchased from Bachem (Bubendorf, Switzerland); cross-linked CRP was obtained from CambCol (Cambridge, United Kingdom); the PAR4 agonist AYPGKF was from Bio-Techne (Minneapolis, Minnesota, United States). Fluorescein isothiocyanate (FITC)-labeled PAC1 monoclonal antibody (mAb) against activated human integrin αIIbβ3 was obtained from BD Bioscience (Franklin Lakes, New Jersey, United States), AF647-labeled anti-human CD62P mAb came from BioLegend (San Diego, California, United States). Dimethyl BAPTA acetoxymethyl ester (DM-BAPTA AM) from Molecular Probes (Eugene, Oregon, United States). Poly-L-lysine hydrobromide (PLL) was from Polysciences (Warrington, Pennsylvania, United States).

The common PKC stimulator PMA and PKC inhibitors, RO-318425 (bisindolylmaleimide X), rottlerin, ML-099, ML-314, PKCθ inhibitor (PKCθ-IN), and glutaraldehyde were from Sigma-Aldrich (St. Louis, Michigan, United States). From the same company came MRS-2179 and apyrase. PKC inhibitor GF109203X (bisindolylmaleimide 1) was obtained from Bio-Techne, and Gö6976 was from MedChem Express (Monmouth Junction, New Jersey, United States). Glycogen synthase kinase-3 inhibitor IX (GSK3-IN), thapsigargin, and the stable ADP analog methylthio-adenosine-diphosphate (Me-S-ADP) came from Santa Cruz Biotechnologies (Dallas, Texas, United States). PI3Kα inhibitor TGX-221 was from the Baker Heart and Diabetes Institute (Melbourne, Victoria, Australia); ticagrelor (AZD6140) from AstraZeneca R&D (Mölndal, Sweden).

### Blood Collection

Human blood was collected from healthy volunteers, after full informed consent according to the Declaration of Helsinki. Approval for the studies was obtained from the local Medical Ethics Committee (METC 10-30-023, Maastricht University). None of the subjects had used antiplatelet medication for at least 2 weeks. Venous blood was collected from an antecubital vein into 3.2% trisodium citrate VACUETTE tubes (Greiner Bio-One, Alphen a/d Rijn, The Netherlands). The first mL of blood was discarded to avoid the presence of tissue factor traces.

### Preparation of Washed Platelets

Platelet-rich plasma (PRP) and washed platelets were prepared, basically as described before. 15 In brief, blood samples were centrifuged at 190 g for 15 minutes. The PRP was carefully collected without taking the buffy coat. After addition of 10 vol% ACD medium (80 mM trisodium citrate, 52 mM citric acid, and 180 mM glucose), platelets in the PRP were spun down in 2 mL Eppendorf tubes at 1700g for 2 minutes. Plasma was removed and the tubes were held upside down for 1 minute to remove remaining plasma. Platelet pellets then were resuspended into 1 mL of HEPES buffer pH 6.6 (136 mM NaCl, 10 mM glucose, 5 mM HEPES, 2.7 mM KCl, 2 mM MgCl _2_ , apyrase at 0.1 units ADPase/mL, and 0.1% bovine serum albumin). After addition of 6.6 vol% ACD, the tubes were recentrifuged, and the washed pelleted platelets were resuspended into 1 mL HEPES buffer pH 7.45 (10 mM HEPES, 136 mM NaCl, 2.7 mM KCl, 2 mM MgCl _2_ , 0.1% glucose, and 0.1% bovine serum albumin). Note that apyrase during the isolation procedure was added to retain platelet responses to ADP and ATP. 20 Platelet count was adjusted to 50 × 10 ^9^ /L for flow cytometry and to 100 × 10 ^9^ /L for microscopy. Blood cells were counted with Sysmex XN-9000 analyzer (Sysmex, Cho-ku, Kobe, Japan).

### Dual Labeling Flow Cytometry

Washed platelets (50 × 10 ^9^ /L) in HEPES buffer pH 7.45 containing 2 mM CaCl _2_ were stimulated for 10 or 20 minutes with CRP (2.5–5 mg/mL), thrombin (2.5–5 nM), TRAP6 (7.5–15 µM), AYPGKF (50 µM), PMA (50 nM), thapsigargin (50 nM), Me-S-ADP (1.0 µM), or indicated combinations. Platelets were either pre- or posttreated with inhibitors, described below. Datasets were analyzed to compare effect of (ant)agonist to specific (day) control samples. Apyrase (0.1 units ADPase/mL) was present, where indicated. For flow cytometry, FITC-PAC1 mAb (1:20) and AF647 anti-P-selectin mAb (1:20) were added at 10 minutes before measurements. Platelet samples were analyzed at least in triplicate. Per analysis, 5,000 events were measured using a BD Accuri C6 flow cytometer (BD Bioscience, Franklin Lakes, New Jersey, United States). 21 Using the manufacturer's software, percentages of positively stained platelets (gating according to forward/side scatter plots) were determined; threshold levels were set at 1 to 2% for unstimulated controls.

### Use of Signaling Inhibitors

Platelets were pre- or posttreated with a panel of signaling pathway inhibitors ( Table 1 ), at previously determined optimal inhibitory concentrations. Regarding PKC (isoform) modulation, 15 17 the nonselective PKC inhibitors RO-318425 (10 µM) and GF109203X (10 µM) were used. 22 Furthermore, the selective PKC isoform inhibitors: Gö6976 targeting PKCα/β > ε (1.0–10 µM); rottlerin targeting PKCδ > α/β (10 µM); or PKCθ-IN targeting PKCθ > δ > α/β (2.5 µM). Used for inhibition of PI3Kβ > α > δ was TGX-221 (0.5 µM) 18 ; for [Ca ^2+^ ] _i_ chelation DM-BAPTA AM (50 µM) 23 ; for inhibition of GSK3α/β the compound GSK3-IN (2.0 µM). 24 The G protein-dependent β-arrestin inhibitor ML-314 was used by convention at 20 µM. 25 26 As a β-arrestin biased allosteric modulator, 27 ML-314 will affect platelets by suppression of G protein-coupled receptor (GPCR) signaling. In addition, ML-314 functions as a neurotensin agonist, 28 which is considered to be irrelevant for platelets. As Ras-related GTPases are critical for full ITAM-induced platelet activation, 29 we also checked the inhibitor of Ras/Rac GTPases (Rac1, Cdc42, etc.) ML-099 at 10 µM. 30 Platelet ADP receptors were blocked with the P2Y _12_ inhibitor ticagrelor (1.0 µM) 31 or the P2Y _1_ inhibitor MRS-2179 (50 µM). 32 Compounds were added to platelets at 10 minutes before or alternatively at 10 minutes after addition of agonist. In the latter case, incubations were continued for another 10 minutes before measurement.

**Table 1:** Pharmacological compounds used for this study

Compound	Target	Explanation	Refs.
Protein kinase C isoforms			
RO-318425	c + nPKC	General PKC inhibitor	15
GF109203X	c + nPKC	General PKCa/b/d/e (GSKα/β)	15 21
Gö6976	cPKC	PKCa/b > e	15
PKCq-IN	nPKC	PKCq > d > a/b	15
Rottlerin	nPKC	PKCd > a/b	15
Other pathways			
DM-BAPTA	[Ca ^2+^ ] _i_	Chelates [Ca ^2+^ ] _i_ when AM removed	22
GSK3-IN	GSK3α/β	GSK3α/β	23
ML-099	SMG	Ras/Rac GTPase activator	26
ML-314	β-arrestins	GPCR β-arrestin inhibitor	24 25
TGX-221	PI3Kβ	PI3Kβ > >PI3Kα > PI3Kδ	18
Thapsigargin	SERCA2b	Ca ^2+^ rise due to SERCA inhibition	38
Autocrine mediators			
Apyrase	ADP	Degrades autocrine ATP, ADP	
MRS-2179	P2Y _1_	ADP receptor inhibitor	28
Ticagrelor	P2Y _12_	ADP receptor inhibitor (ENT1)	27

Abbreviations: AM, acetoxymethyl ester; ADP, adenosine diphosphate; ATP, adenosine triphosphate; GPCR, G protein-coupled receptor; GSK, glycogen synthase kinase; PI3K, phosphoinositide 3-kinase; PKC, protein kinase C.

### Microscopy

Washed platelets (100 × 10 ^9^ /L) in HEPES buffer pH 7.45 containing 2 mM CaCl _2_ were left unstimulated or were stimulated with CRP (5 µg/mL) or TRAP6 (15 µM) for 10 minutes and then posttreated with RO-318425 (10 µM), GF109203X (3.0 µM), or ticagrelor (1.0 µM) for 10 minutes. Alternatively, platelets were pretreated with the above indicated inhibitors for 10 minutes and then stimulated with CRP or TRAP6 for 10 minutes. The cells were then fixed with 1.5% glutaraldehyde in 0.1 M phosphate buffer pH 7.4 for 15 minutes. 10 The fixed platelets were plated on PLL-coated coverslips for 1.5 hours and rinsed with HEPES buffer pH 7.45. Platelet morphological responses were assessed using an inverted light transmission microscope with 63× objective (Leica DFC 3000 G, Wetzlar, Germany).

### Heatmap Clustering and Statistics

Heatmaps were generated using the program R (version 4.2.1). Univariate scaling was applied to all parameters, as before. 33 In brief, for rainbow-colored heatmaps, raw data were normalized versus maximal values per subject and then rescaled 0 to 10. Relative inhibitor effects were scaled accordingly and are represented as mean values. 34 For effect heatmaps (green-red colors), percentual inhibitor effects were first calculated per subject, and relative inhibitor results were scaled (0–100%) and represented as mean. Primary data were compared by a two-tailed (paired) Student's *t* -test. For multiple comparisons, Benjamini–Hochberg corrections were applied. For image analysis data, a two-sided chi-square test was used. *p* -Values < 0.05 were considered as statistically significant. GraphPad Prism 8 software was used for statistical analysis.

## Results

### Reversibility of Integrin αIIbβ3 Activation upon Glycoprotein VI or Protease-Activated Receptor Stimulation

Considering preliminary evidence for agonist-induced desensitization of platelets, 35 we systematically monitored how key agonists evoked platelet activation changes on the shorter (i.e., 10 minutes) and the longer (20 minutes) terms. Herein, we used submaximal and lower doses of CRP (stimulating GPVI), TRAP6 (for PAR1), AYPGKF (for PAR4), as well as thrombin (for PAR1/4). We thus checked the agonists' ability to induce integrin αIIbβ3 activation and P-selectin degranulation, using flow cytometry and FITC-PAC1 mAb directed against the activated epitope of αIIbβ3 plus AF647 anti-P-selectin mAb, monitoring dense granule secretion.

**Fig. 1:**
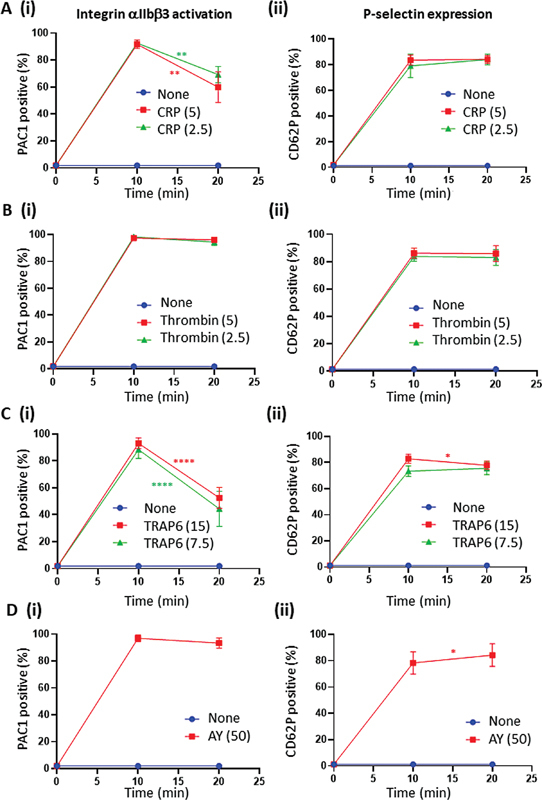
Time-dependent effects of GPVI- and PAR-induced integrin αIIbβ3 activation and P-selectin expression. Washed platelets were stimulated for 10 or 20 minutes with indicated doses of CRP (μg/mL), thrombin (nM), TRAP6 (μM) or AYPGKF (AY, μM). The platelet samples were then labeled with FITC-PAC1 mAb and AF647 anti-CD62P mAb for active intgrins and P-selectin expression, respectively, for analysis by flow cytometry. Shown are percentages of positive platelets in response to CRP ( **A** ), thrombin ( **B** ), TRAP6 ( **C** ), or AYPGFK ( **D** ). Representative histograms are shown in Suppl. Figure 1. Data are means ± SD ( *n* = 3–6); * *p* < 0.05, ** *p* < 0.01, *** *p* < 0.001, **** *p* < 0.0001 for 10 min *vs.* 20 min ( *t* -test).

Platelet stimulation via GPVI with CRP, dose independently, caused a partial (5 μg/mL: 92 → 60%; 2.5 μg/mL: 93 → 70%) transiency of PAC1 mAb binding, reflecting αIIbβ3 inactivation, while P-selectin expression remained high over 20 minutes ( Fig. 1A i–ii, representative histograms are shown in Supplementary Fig. S1 ). The latter observation agrees with the known irreversibility of granular exocytosis. 36 Transiency of the integrin activation was similar in size, when calculated as percentage of positive platelets or as mean fluorescence intensity (data not shown).

Stimulation of platelets with the PAR agonists resulted in specific differences. After longer-term activation with thrombin (20 minutes), αIIbβ3 activation remained high ( Fig. 1B ). In contrast, stimulation with PAR1 agonist TRAP6 first led to high αIIbβ3 activation, which then substantially declined (15 mM: 93 → 53%; 7.5 mM: 88 → 44%, Fig. 1C ). On the other hand, PAR4 stimulation with AYPGKF led to permanently high αIIbβ3 activation markers ( Fig. 1D ). With all PAR agonists, P-selectin expression remained high upon prolonged incubation. These results hence point to reversibility of the integrin αIIbβ3 activation process, which is most prominent in response to PAR1 stimulation.

**Fig. 2:**
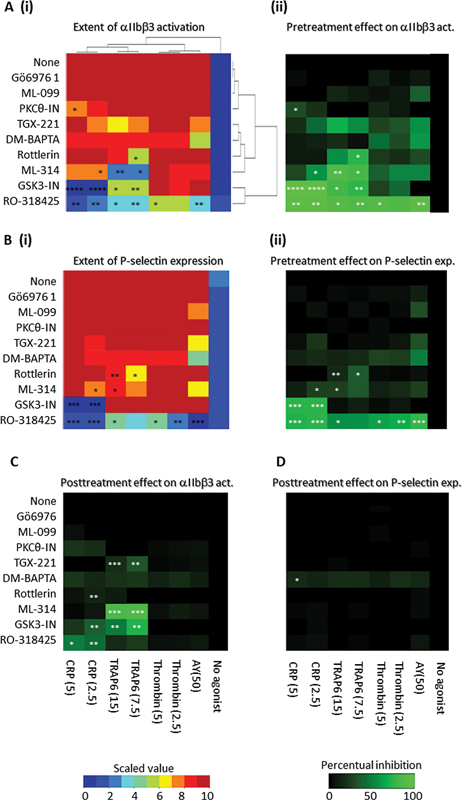
Effects of signaling inhibitors on GPVI- and PAR-induced αIIbβ3 activation and P-selectin expression. Washed platelets were pretreated for 10 minutes with vehicle (none) or optimized doses of Gö6976 (1 µM), ML-099 (10 µM), PKCθ-IN (2.5 µM), TGX-221 (0.5 µM), DM-BAPTA (50 µM, 30 minutes), rottlerin (10 µM), ML-314 (20 µM), GSK3-IN (2 µM) or RO-318425 (10 µM). Subsequently, the platelet samples were left unstimulated or stimulated with CRP (5 or 2.5 µg/mL), TRAP6 (15 or 7.5 µM), thrombin (5 or 2.5 nM) or AYPGKF (50 µM) for 10 mins. Using flow cytometry, αIIbβ3 activation (% PAC1 positive) and P-selectin expression (% CD62P positive) were assessed, as in Figure 1. Data were scaled 0-10 from unstimulated to maximal activation marker, based on mean values ( *n* = 3-6). ( **A** ) Unsupervised clustering of scaled values of αIIbβ3 activation ( **i** ), and percentage change by indicated signaling inhibitor ( **ii** ). The resulting ordering of agonists and inhibitors was used for all heatmap presentations. ( **B** ) Corresponding heatmaps of extent of P-selectin expression ( **i** ) and differential percentage change by indicated signaling inhibitor ( **ii** ). For the posttreatment condition, platelets were left unstimulated or stimulated for 10 minutes with CRP (5 or 2.5 µg/mL), TRAP6 (15 or 7.5 µM), thrombin (5 or 2.5 nM) or AYPGKF (50 µM). After 10 minutes, the cells were posttreated with vehicle (none) or indicated signaling inhibitors, as mentioned above. After 10 minutes, integrin αIIbβ3 activation (% PAC1 positive) and P-selectin expression (% CD62P positive) were measured by flow cytometry. Shown are heatmaps of procentual changes in positive platelets versus no inhibitor for αIIbβ3 activation ( **C** ) or P-selectin expression ( **D** ). Color bars refer to scaling used. Means ( *n* ≥ 3); * *p* < 0.05, ** *p* < 0.01, *** *p* < 0.001 for inhibitor vs. control condition (none) without inhibitor at endpoint ( *t* -test).

To determine which intracellular signaling pathways control initial αIIbβ3 activation induced by these agonists, we used a panel of nine pharmacological inhibitors, selected from the literature at published optimal inhibitory concentrations ( Table 1 ). This agonist panel was used to compare platelet stimulation with CRP, TRAP6, thrombin, and AYPGKF. Integrin αIIbβ3 activation and P-selectin expression were again measured after 10 minutes. Unsupervised clustering analysis of the results provided a heatmap, which illustrated that the overall inhibitory effect per agonist was larger for CRP (2 × ) and TRAP6 (2 × ) than for thrombin and AYPGKF ( Fig. 2A i). The antagonist clustering followed the overall inhibitory effect size, i.e., smallest for Gö6976 (no inhibition, PKCα/β > ε) and largest for RO-318425 (high inhibition, nonselective PKC). The inhibitor effects on P-selectin expression were strikingly similar ( Fig. 2B i). When calculated as percentual inhibition ( Fig. 2A, B ii), the inhibition of RO-318425 (general PKC) on αIIbβ3 activation and P-selectin expression extended to all agonists, whereas that of GSK3α/β inhibitor were confined to CRP and TRAP6. Other compounds showing significant inhibitory effects with distinct agonists were the β-arrestin inhibitor ML-314 (CRP, TRAP6), rottlerin (PKCδ > α/β) (TRAP6), and PKCθ-IN (CRP). Nonsignificant effects were obtained with TGX-221 (PI3Kβ blocker); DM-BAPTA (cytosolic Ca ^2+^ chelation); Gö6976 (PKCα/β blocker); and ML-099 (Ras-type blocker).

To further assess whether secondary signaling inhibition was also able to revert the integrin αIIbβ3 activation, we added this panel of compounds at 10 minutes after the agonists ( Fig. 2C ). Now, RO-318425, GSK3-IN and ML-314 significantly downregulated the extent of activated αIIbβ3 induced by CRP or TRAP6 ( Fig. 2D ). Weakly, but significantly inhibitory were rottlerin, reverting after CRP (2.5 μg/mL: 12% reduction) and TGX-221 reverting after TRAP6 (7.5 mM: 32%; 15 mM: 17% reduction). None of the compounds did reverse responses to thrombin or AYPGKF. Jointly, these results indicate high integrin activation process versatility.

### Role of Combined Protein Kinase C Isoforms in Continued Integrin αIIbβ3 Activation

**Fig. 3:**
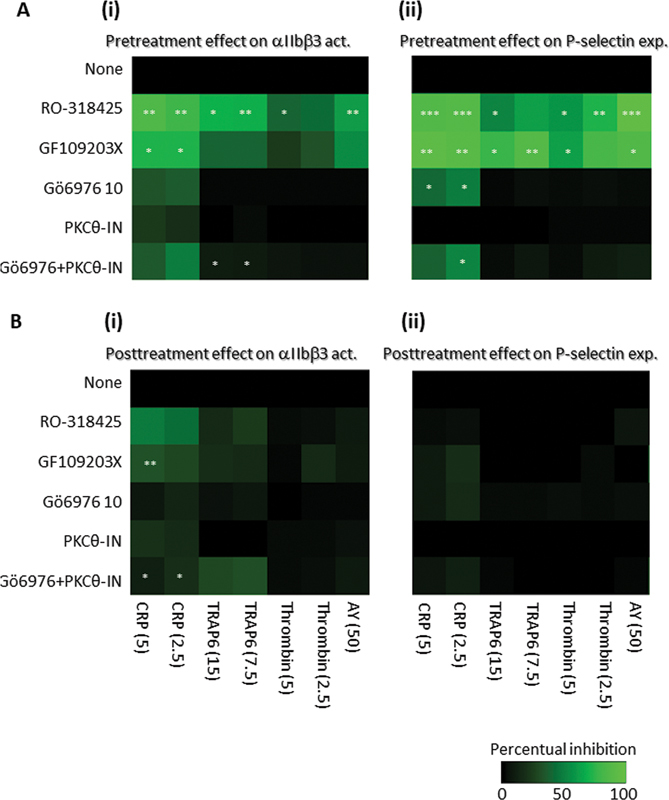
Effect of PKC isoforms on GPVI- and PAR-induced integrin αIIbβ3 activation and P-selectin expression. For the pretreatment condition, platelets in suspension were pretreated for 10 mins with vehicle (none) or optimized doses of RO-318425 (10 µM), GF109203X (10 µM), Gö6976 (10 µM), PKCθ-IN (2.5 µM) or a mixture of Gö6976 (10 µM) and PKCθ-IN (2.5 µM). The cells were then stimulated for 10 minutes with agonists ( Fig. 2 ). For the posttreatment condition, the platelets were stimulated with indicated agonists. After 10 minutes, vehicle (none) or indicated signaling inhibitors were added. Activation markers were measured 10 minutes later by flow cytometry. Shown are heatmaps of procentual changes in positive platelets versus no inhibitor for αIIbβ3 activation ( **Ai** , **Bi** ) and P-selectin expression ( **Aii** , **Bii** ). Color bar refers to scaling used. Means ( *n* ≥ 3); * *p* < 0.05, ** *p* < 0.01 for inhibitor vs. control condition (none) without inhibitor at endpoint ( *t* -test). For graphs of raw data (PAC positive, % of control), see Supplementary Fig. 2 .

To further elucidate the overall role of PKC in integrin inactivation, we tested the general PKC inhibitor, GF109203X, which had a similar antagonizing effect as RO-318425 on CRP- and TRAP6-dependent αIIbβ3 activation (PAC1 mAb binding), upon both pre- and posttreatment ( Fig. 3A, B , graphs of raw data are shown in Supplementary Fig. S2 ). In addition, we combined the PKCα/β inhibitor Gö6976 with PKCθ inhibitor PKCθ-IN to check for synergy between the conventional and novel PKC isoforms. 16 Here, we observed a reduced αIIbβ3 activation in case of pretreatment (TRAP6 15 mM, *p* = 0.022; TRAP6 7.5 mM, *p* = 0.021) or posttreatment (CRP 15μg/mL, *p* = 0.019; CRP 7.5 μg/mL, *p* = 0.013 ( Fig. 3A-B i).

**Fig. 4:**
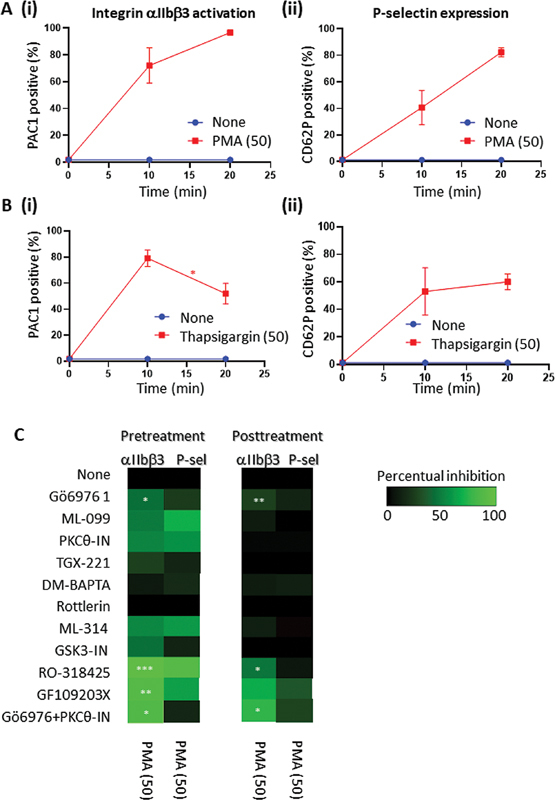
Roles of PKC activation and elevated Ca2+ on integrin αIIbβ3 activation and P-selectin expression. Flow cytometry measurements of washed platelets, stimulated for 10 or 20 minutes with 50 nM PMA ( **A** ) or 50 nM thapsigargin ( **B** ). Integrin αIIbβ3 activation ( **i** ) and P-selectin expression ( **ii** ) were measured, as for Figure 1. Shown are percentages of positive platelets. ( **C** ) Effect of platelet pretreatment (-10 minutes) or posttreatment (+10 minutes) with indicated inhibitors (doses as in Fig. 2) on PMA-induced activation markers. Heatmaps provide changes of % positive platelets vs. no inhibitor. Means (± SD, A,B) ( *n* ≥ 3); * *p* < 0.05, ** *p* < 0.01, *** *p* < 0.001 for 10 min *vs.* 20 min (A, B) or for inhibitor vs. control condition (none) without inhibitor at endpoint ( *t* -test). For representative histograms, see Supplementary Fig. 3 .

To check for possible transiency of the PKC activity, we treated the platelets with the pan-PKC agonist PMA. 15 By itself, PMA induced a slow but continued integrin αIIbβ3 activation ( Fig. 4A i, for representative histograms, see Supplementary Fig. S3 ), along with P-selectin expression ( Fig. 4A ii). On the other hand, the Ca ^2+^ -mobilizing agent thapsigargin—with only limited PKC activity—induced an αIIbβ3 activation that was transient over time (79 → −59%, Fig. 4B i–ii).

To determine the involvement of specific PKC isoforms, we applied the same panel of inhibitors in either pre- or posttreatment. When pretreated, significant inhibition of αIIbβ3 activation could be obtained with RO-318425, G109203FX, and the combination of Gö6976 + PKCθ-IN, i.e., by combined inhibition of conventional and novel PKC isoforms ( Fig. 4C ). Other compounds were insignificantly effective. The PMA-induced P-selectin expression was affected to a lesser extent. Similarly, posttreatment with the general PKC inhibitors at 10 minutes after PMA did suppress the activation of αIIbβ3.

### Stabilizing Role of (Autocrine) ADP via P2Y _12_ Receptors in Integrin αIIbβ3 Activation

**Fig. 5:**
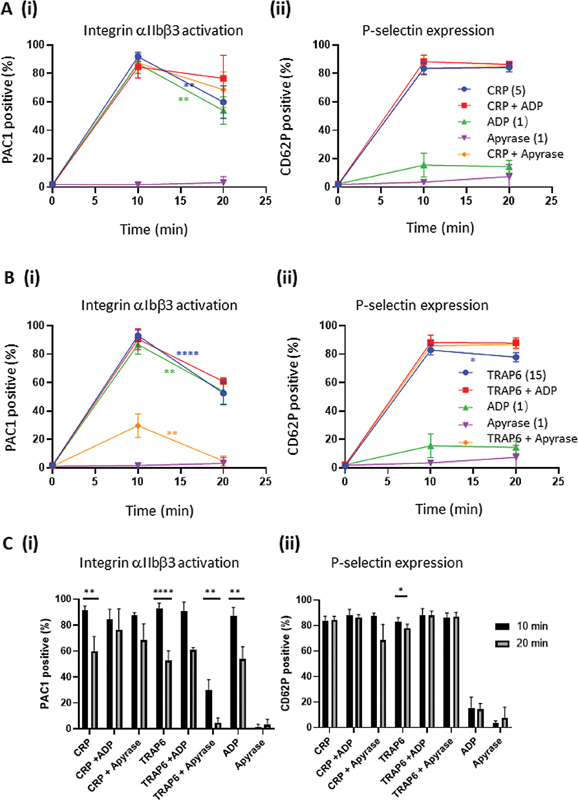
Time effects of ADP receptor-dependent integrin αIIbβ3 activation and P-selectin expression. Platelets were stimulated for 10 or 20 minutes as indicated with CRP (5 μg/mL), TRAP6 (15 μM) and/or ADP (1 μM). Samples were pre-incubated with ADP-degrading apyrase (1 U/mL). Integrin αIIbβ3 activation ( **i** ) and P-selectin expression ( **ii** ) were measured, as for Fig. 1 . Shown are percentage of positive platelets in response to CRP and/or ADP ( **A** ), or to TRAP6 and/or ADP ( **B** ), with additional quantification ( **C** ). Means ± SD ( *n* ≥ 3); * *p* < 0.05, ** *p* < 0.01, **** *p* < 0.0001 for 10 min vs. 20 min ( *t* -test). Representative histograms in Supplementary Figs. 4–5 .

Considering that the platelet ADP receptors contribute to continued platelet aggregation with several agonists, 4 we also investigated the role of autocrine released ADP in the long-term integrin activation. For the two agonists showing reversibility, i.e., CRP and TRAP6, we thus monitored the effects of added Me-S-ADP (a stable ADP form) or apyrase (degrading autocrine ADP). With CRP, we found that the Me-S-ADP addition—but not Me-S-ADP alone—resulted in long-term (20 minutes), essentially irreversible αIIbβ3 activation ( Fig. 5A i) but without effect on the already maximal P-selectin expression ( Fig. 5A ii). For TRAP6, we measured that the Me-S-ADP addition was unable to prevent reversibility of the αIIbβ3 activation process, whereas apyrase addition markedly reduced the extent and duration of integrin activation, but not of P-selectin expression ( Fig. 5B i–ii). Quantitative comparison for the two agonists underpinned the more transient and sensitive αIIbβ3 activation (not P-selectin expression) with TRAP6 than with CRP ( Fig. 5C , representative histograms in Supplementary Figs. S4 and S5 ). Together, these results indicated a more important contribution of autocrine ADP for TRAP6 than for CRP responses.

**Fig. 6:**
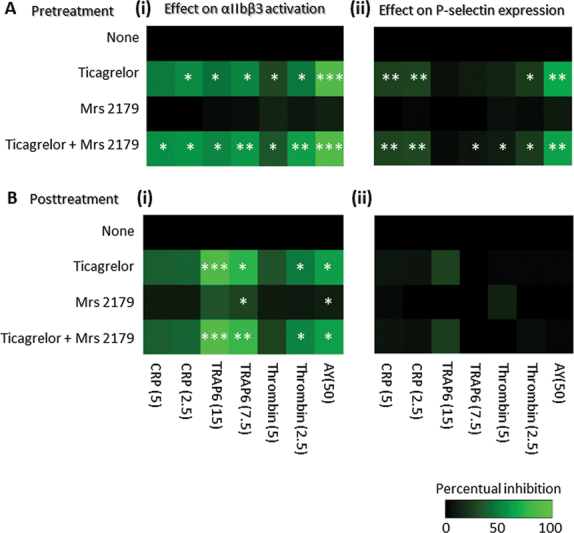
Role of ADP on GPVI- and PAR-induced integrin αIIbβ3 activation and P-selectin expression. In the pretreatment condition ( **A** ), platelets were incubated for 10 minutes with vehicle (none) or optimized doses of ticagrelor (1 µM) and/or MRS-2179 (50 µM). The cells were subsequently stimulated with CRP (5 or 2.5 µg/mL), TRAP6 (15 or 7.5 µM), thrombin (5 or 2.5 nM) or AYPGKF (50 µM). In the posttreatment condition ( **B** ), the platelets were left unstimulated or stimulated with the same agonists. After 10 minutes, the platelets were posttreated with vehicle (none) or ticagrelor and/or MRS-2179. Flow cytometric detection was performed 10 minutes later, as in Fig. 1 . Shown are heatmaps of extent of αIIbβ3 activation ( **i** ), and P-selectin expression ( **ii** ). Means ( *n* = 3–4); * *p* < 0.05, ** *p* < 0.01, *** *p* < 0.001 for inhibitor vs. control condition (none) without inhibitor at endpoint ( *t* -test). For graphs of raw data (PAC positive, % of control), see Supplementary Fig. 6 .

To distinguish between the two ADP receptors, we repeated the CRP and TRAP6 experiments with added ticagrelor (P2Y _12_ antagonist) or MRS-2179 (P2Y _1_ antagonist) either before or after platelet stimulation with agonist. We employed ticagrelor as antagonist, given its regular use in the clinic. Platelet pretreatment with ticagrelor, but not with MRS-2179, significantly suppressed the integrin activation process in response to CRP or TRAP6 and to a lesser degree to thrombin ( Fig. 6A i, for graphs of raw data see Supplementary Fig. S6 ). With ticagrelor also P-selectin expression was affected to a certain extend ( Fig. 6A ii). Strikingly, even posttreatment of ticagrelor after 10 minutes reduced the integrin activation with CRP (not significant), TRAP6, and thrombin (significant ( Fig. 6B i), but left P-selectin expression unaffected ( Fig. 6B ii).

### Platelet Shape and Content Change on Pre- and Posttreatment

**Fig. 7:**
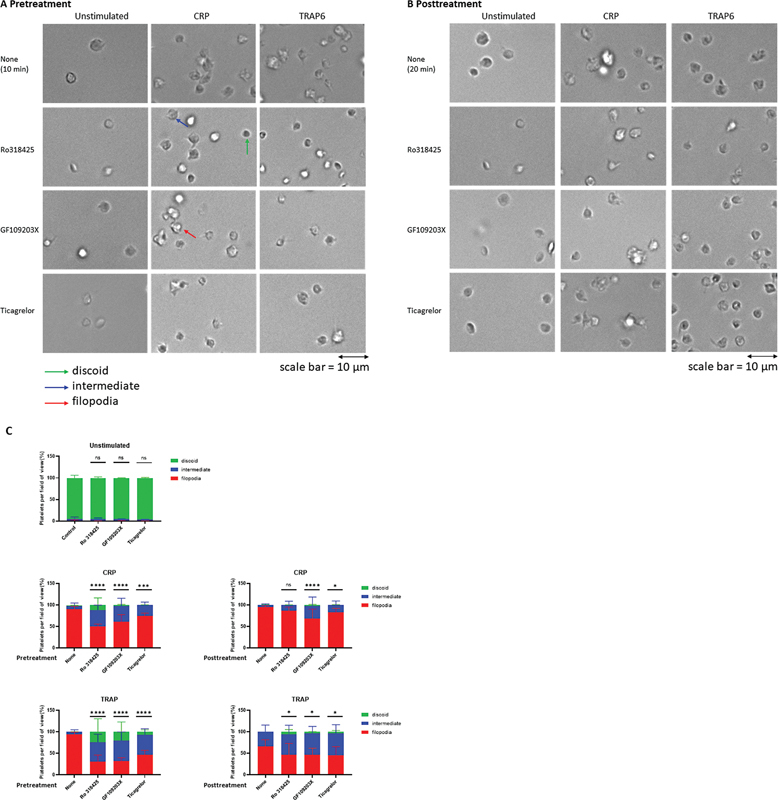
GPVI- and PAR1-induced changes in platelet morphology. Washed platelets were treated with RO-318425 (10 µM), GF109203X (3 µM), or ticagrelor (1 µM) for 10 minutes before ( **A** ) or after ( **B** ) stimulation with 5 µg/mL CRP-XL or 15 µM TRAP6 (or vehicle as a control) for 10 minutes. Platelet morphology was imaged using an inverted light transmission microscope with 63x objective, scale bar = 10 µm, all images are the same size. ( **C** ) Morphology was assessed for percentage of platelets with disc-shape (‘discoid’), intermediate phenotype (“intermediate”), or formed filopodia and lamellipodia (“filopodia”). Means ± SD ( *n* = 3–4); Chi-square test, * *p* < 0.05, ** *p* < 0.01, *** *p* < 0.001, **** *p* < 0.0001 for inhibitor vs. control condition (none) without inhibitor at endpoint.

To further confirm a temporary role of PKC and ADP signaling in platelet responses, we monitored integrin-related morphological changes over time. Using microscopy, we quantified the number of activated platelets with a discoid, resting morphology and with complete or intermediate shape changes by the formation of filopodia. 10 In TRAP6-stimulated platelets, representative images indicated transiency over time ( Fig. 7A ), which was confirmed by quantification of the platelets with filopodia, reducing from 94 to 66% over 20 minutes ( Fig. 7C ). In contrast, CRP-stimulated platelets did not significantly revert their shapes.

Upon preincubation with RO-318425, reduced fractions of 50 and 31% of platelets displayed filopodia in response to CRP and TRAP6, respectively. For pretreatment with GF109203X, the platelets with filopodia are 60 and 32% after stimulation with CRP and TRAP6, respectively. Less effect was seen upon ticagrelor pre-incubation; i.e., reductions to 74% for CRP and 47% for TRAP6. Importantly, also the posttreatment of CRP or TRAP6-stimulated platelets with GF109203X and ticagrelor caused a significant decrease in platelets with filopodia formation ( Fig. 7B, C ).

## Discussion

In this paper, we elucidated the roles of conventional and novel PKC isoforms and of autocrine ADP in sustained platelet integrin αIIbβ3 activation. Under conditions of PKC or ADP inhibition, this process became transient, while we did not observe notable reversibility of P-selectin expression. Most versatile receptors in terms of transiency appeared to be PAR1 (triggered by TRAP6), ADP (by P2Y _12_ ), and GPVI (by CRP), compared with PAR4. In agreement with this, morphological assessment of platelets also showed that PAR1 triggered a transient manner of platelet activation. The reversibility in response to GPVI agonist—although in effect smaller than for PAR1—is remarkable, given the prior evidence for a longer-term “memory” effect of GPVI-stimulated platelets in comparison to PAR1. 35 Although longer term, part of the GPVI-stimulated platelets are still able to reset their activation state. This agrees with the earlier reported restimulating effect of ADP after previous GPVI stimulation. 10

While receptor signaling via PAR1 resulted in reversible integrin activation (i.e., integrin closure), we found that signaling via PAR4 induced a more permanent integrin activation, which also appeared from the different susceptibility to pharmacological inhibitors. These observations are in line with the known concept that PAR1 activation primes for subsequent PAR4 activation.

When comparing GPVI-induced versus PAR1-induced integrin activation (either being reversible), we found the latter is much more reliant on ADP release from dense granules, an aspect that is often not recognized. This implies that PAR1 alone is a relatively weak platelet-activating agonist, while on human platelets the thrombin effect greatly relies on the PAR1–PAR4 axis. This is in accordance with the earlier finding that human platelets after GPVI stimulation are longer in an activation-primed condition than after PAR1 stimulation. 35 After initial activation, the platelet shape can return from filopodia-containing to discoid. 10 The currently observed PKC- and ADP-dependent reversion of this morphological change indicates that reversibility of the αIIbβ3 activation state is not an isolated event but extends to cytoskeletal changes in platelets as well.

In terms of signals contributing to a permanent integrin αIIbβ3 activation, among agonists (CRP, TRAP6), the data point to major contributions of isoforms of PKC and also to a lesser extent of isoforms of GSK3α/β and β-arrestin (inhibited by ML-314). The observed inhibitory action of ML-314 on (low) CRP- and TRAP6-induced integrin activation, with a clear contribution of ADP, in our view provides an indication of the inhibitory mechanism. Its most likely effect on platelets is suppression of the GPCR signaling via one or more of the P2Y receptors. However, more work needs to be done to confirm this idea. Unexpectedly, the contribution of PI3Kβ (inhibited by TGX-221) was small. In comparison, Wu et al have shown that the combined blockage of PAR4 and PI3K changes thrombin-elicited platelet aggregation from an irreversible to a reversible event. 37 Therefore, and based on our findings, this suggests that an early activation of PAR1 by thrombin generates insufficient PKC activity to keep αIIbβ3 in an activated state.

In human platelets, the overall role of PKC is jointly mediated by the conventional, Ca ^2+^ -dependent PKC isoforms α/β (inhibited by Gö6976, RO-318425 and GF109203X) and the novel, Ca ^2+^ -independent PKC isoforms δ/θ (inhibited by rottlerin, PKCθ-IN, RO-318425, and GF109203X). 15 16 17 38 In collagen-induced thrombus formation under flow, the conventional isoforms, PKCα and PKCβ, positively contribute to α-granule secretion and aggregate buildup, 15 17 whereas the novel isoforms, PKCθ and PKCδ, rather act in a suppressive way. 15 39 Our current data indicate that the inhibition of all PKC isoforms with RO-318425 or GF109203X greatly reduces the ability to activate integrin αIIbβ3. In another paper by our group, we demonstrate that under the same conditions GF109203X near completely blocks the GPVI-induced and PKC-dependent phosphorylation of Btk S180 (as a marker) and furthermore of Syk S297 and MEK1/2 S219/S221 (Zhang et al, unpublished data). Interestingly, also the combination of compounds Gö6976 and PKCθ-IN affected the agonist-induced or PMA-induced αIIbβ3 activation. Together, this pointed to additive contributions of the conventional and novel PKC isoforms in a time-dependent control of integrin αIIbβ3 activity.

The observed effect of GSK3α/β inhibition in CRP-stimulated platelets can be explained by phosphorylation of GSK3α as a requirement for the inhibition of GPVI-mediated platelet activation. 40 The moderate suppressive effects of β-arrestin inhibitor ML-314 (with TRAP6 > CRP) are interesting, because β-arrestins may terminate PAR1-induced signaling in platelets and other cells. 41 42 Whereas the PI3Kα pathway is considered as one of the players for prolonged platelet aggregation, 4 37 the present experiments gave no more than limited effects of TGX-221 in reducing the αIIbβ3 activity; significance was only obtained upon posttreatment after PAR1 stimulation.

Limitations of our study are the potential off-target effects of the used inhibitors. In this regard, we have taken advantage of the screening paper by Anastassiadis et al, where the authors used large-scale profiling assays to identify the kinase spectrum targeted by pharmacological inhibitors. 43 It should be further noted that the P2Y _12_ inhibitor of choice, ticagrelor, can also act as an adenosine reuptake inhibitor, resulting in elevated cAMP levels known to lead to integrin closure. 44

Altogether, as presented in the graphical summary of Supplementary Fig. S7 , we conclude that particularly PKC and autocrine ADP signaling contribute to a persistent integrin αIIbβ3 activation in PAR1/GPVI > PAR4-stimulated platelets. These findings on pathway-dependent permanency of platelet integrin αIIbβ3 activation are critical for optimization of the effectivity of antiplatelet treatment approaches.

## Notes

**What is known about this topic?**

- The integrin αIIbβ3 conformational change, underlying agonist-induced platelet activation and aggregation, is considered reversible.
- Autocrine released ADP sustains and several PKC isoforms contribute to the aggregation process.

**What does this paper add?**

- Reversibility of integrin αIIbβ3 activation is pathway-dependent with the contribution of PKC and autocrine ADP signalling being larger upon PAR1/GPVI > PAR4 stimulation.
- Comparing GPVI-induced vs. PAR1-induced integrin activation (either being reversible), the latter is much more reliant on ADP release from dense granules.
- The PKC- and ADP-dependent reversion of the platelet morphological change indicates that reversibility of the αIIbβ3 activation state is not an isolated event, but extends to cytoskeletal changes in platelets as well.
