# Supplementary material for: Platelet Activation Pathways Controlling Reversible Integrin αIIbβ3 Activation
Source: TH Open. 2024 Jun 22;8(2):e232–42. doi: 10.1055/s-0044-1786987 (PMC11193594; doi:10.1055/s-0044-1786987)
Supplement: Supplementary file 1 — Supplementary Material [file 10-1055-s-0044-1786987-s24010002.pdf]

Platelet activation pathways controlling reversible integrin αIIbβ3 activation

Jinmi Zou[1,2], Siyu Sun[1,2], Ilaria De Simone[1], Hugo ten Cate[2], Philip G. de Groot[1], Bas de Laat[1], Mark Roest[1], Johan W.M. Heemskerk[1], Frauke Swieringa[1]

1. Synapse Research Institute, Maastricht, Koningin Emmaplein 7, 6217 KD Maastricht, The Netherlands;

2. Departments of Biochemistry and Internal Medicine, Maastricht University Medical Center+, Maastricht, The Netherlands.

Methods

Blood collection

Human blood was collected from healthy volunteers, after full informed consent according to the Declaration of Helsinki. Approval for the studies was obtained from the local Medical Ethics Committee (METC 10-30-023, Maastricht University). None of the subjects had used antiplatelet medication for at least 2 weeks. Venous blood was collected from an antecubital vein into 3.2% trisodium citrate Vacuette tubes (Greiner Bio-One, Alphen a/d Rijn, The Netherlands). The first mL of blood was discarded to avoid the presence of tissue factor traces.

Preparation of washed platelets

Platelet-rich plasma (PRP) and washed platelets were prepared, basically as described before. In brief, blood samples were centrifuged at 190 g for 15 minutes. The PRP was carefully collected without taking the buffy coat. After addition of 10 vol% ACD medium (80 mM trisodium citrate, 52 mM citric acid and 180 mM glucose), platelets in the PRP were spun down in 2 mL Eppendorf tubes at 1700 g for 2 minutes. Plasma was removed and the tubes were held upside down for 1 minute to remove remaining plasma. Platelet pellets then were resuspended into 1 mL of Hepes buffer pH 6.6 (136 mM NaCl, 10 mM glucose, 5 mM Hepes, 2.7 mM KCl, 2 mM MgCl2, apyrase at 0.1 units ADPase/mL and 0.1% bovine serum albumin). After addition of 6.6 vol% ACD, the tubes were recentrifuged, and the washed pelleted platelets were resuspended into 1 mL Hepes buffer pH 7.45 (10 mM Hepes, 136 mM NaCl, 2.7 mM KCl, 2 mM MgCl2, 0.1% glucose and 0.1% bovine serum albumin). Note that apyrase during the isolation procedure was added to retain platelet responses to ADP and ATP. Platelet count was adjusted to 50×10^9/L for flow cytometry and to 100×10^9/L for microscopy. Blood cells were counted with Sysmex XN-9000 analyzer (Sysmex, Cho-ku, Kobe, Japan).

Dual labeling flow cytometry

Washed platelets (50×10^9/L) in Hepes buffer pH 7.45 containing 2 mM $CaCl_2$ were stimulated for 10 or 20 minutes with CRP (2.5-5 µg/mL), thrombin (2.5-5 nM), TRAP6 (7.5-15 µM), AYPGKF (50 µM), PMA (50 nM), thapsigargin (50 nM), Me-S-ADP (1.0 µM), or indicated combinations. Platelets were either pre-or post-treated with inhibitors, described below. Datasets were analyzed to compare effect of (ant)agonist to specific (day) control samples. Apyrase (0.1 units ADPase/mL) was present, where indicated. For flow cytometry, FITC-PAC1 mAb (1:20) and AF647 anti-P-selectin mAb (1:20) were added at 10 minutes before measurements. Platelet samples were analyzed at least in triplicate. Per analysis, 5,000 events were measured using a BD Accuri C6 flow cytometer (BD Bioscience, Franklin Lakes NJ, USA). Using the manufacturer's software, percentages of positively stained platelets (gating according to forward/side scatter plots) were determined; threshold levels were set at 1-2% for unstimulated controls.

Use of signaling inhibitors

Platelets were pre- or post-treated with a panel of signaling pathway inhibitors, at previously determined optimal inhibitory concentrations. Regarding PKC (isoform) modulation, used were the non-selective PKC inhibitors RO-318425 (10 µM) and GF109203X (10 µM). Furthermore, the selective PKC isoform inhibitors: Gö6976 targeting PKCα/β>ε (1.0-10 µM); rottlerin targeting PKCδ>α/β (10 µM); or PKCθ-IN targeting PKCθ>δ>α/β (2.5 µM). Used for inhibition of PI3Kβ>α>δ was TGX-221 (0.5 µM); for $[Ca^{2+}]i$ chelation DM-BAPTA AM (50 µM); for inhibition of GSK3α/β the compound GSK3-IN (2.0 µM). The G-protein-dependent β-arrestin inhibitor ML-314 was by convention used at 20 µM. As several Ras-related GTPases are critical for a full ITAM-induced platelet activation, we used the inhibitor of Ras/Rac GTPases (e.g., Rac1, Cdc42) ML-099 at 10 µM. Platelet ADP receptors were blocked with the $P2Y_{12}$ inhibitor ticagrelor (1.0 µM) or the $P2Y_1$ inhibitor MRS-2179 (50 µM). Compounds were added to platelets at 10 minutes before or alternatively at 10 minutes after addition of agonist. In the latter case, incubations were continued for another 10 minutes before measurement.

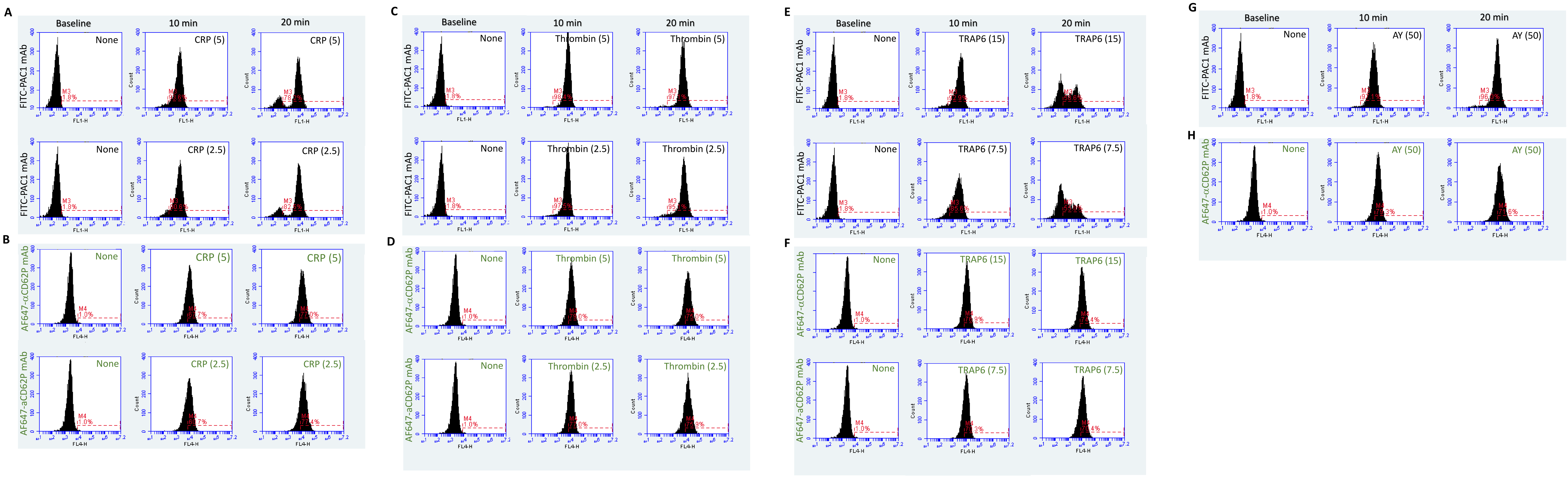

Suppl. Figure 1. Time-dependent effects of GPVI- and PAR-induced integrin αIIbβ3 activation and P-selectin expression. Washed platelets were stimulated for 10 or 20 minutes with indicated doses of CRP (μg/mL), thrombin (nM), TRAP6 (μM) or AYPGKF (AY, μM). The platelet samples were then labeled with FITC-PAC1 mAb and AF647 anti-CD62P mAb for active integrins and P-selectin expression, respectively, for analysis by flow cytometry. Representative histograms are shown for stimulation with CRP (**A,B**), thrombin (**C,D**), TRAP6 (**E,F**), or AYPGFK (**G**).

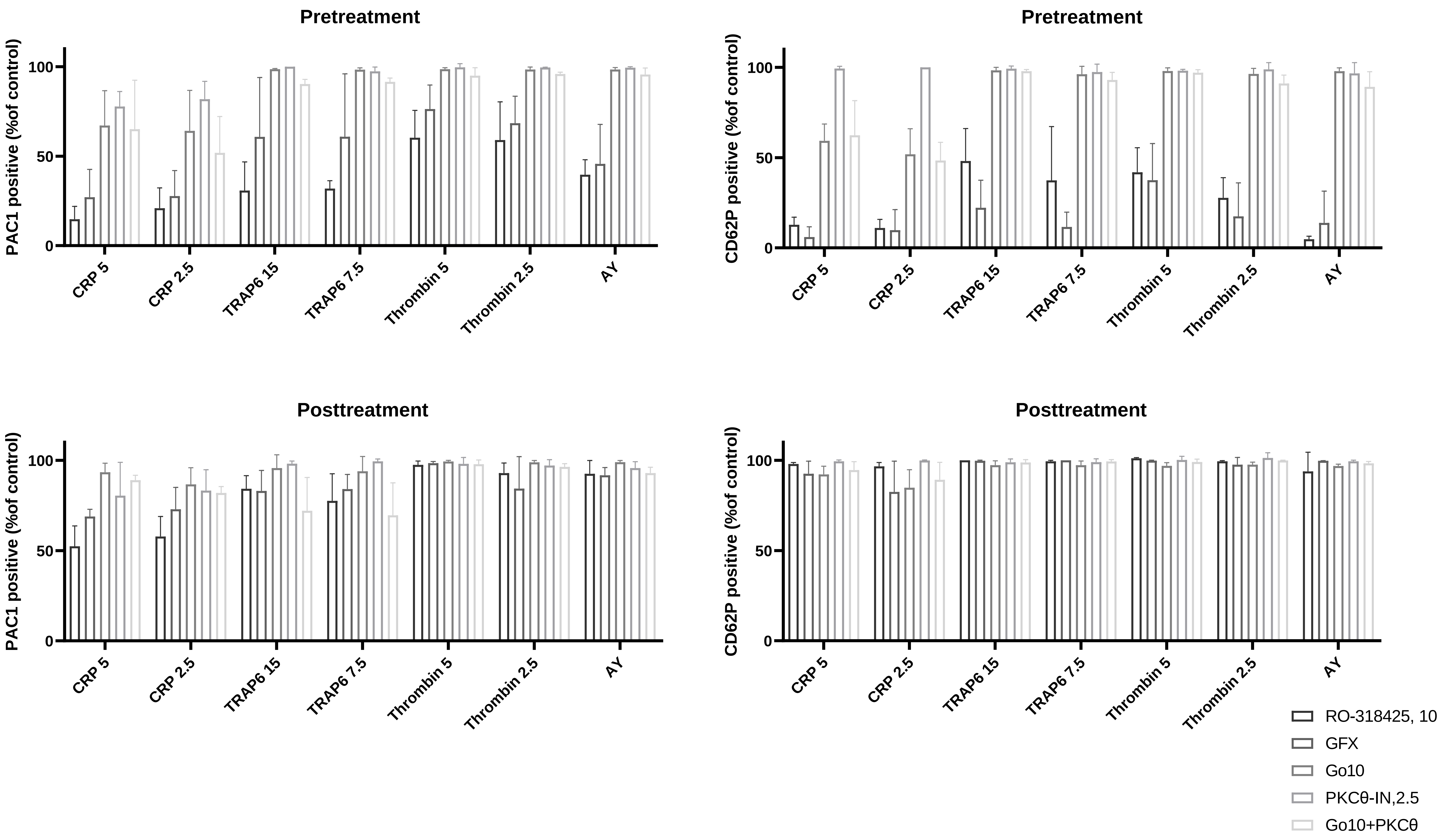

Suppl. Figure 2. Effect of PKC isoforms on GPVI- and PAR-induced integrin αIIbβ3 activation and P-selectin expression. For the pretreatment condition, platelets in suspension were pretreated for 10 minutes with vehicle or optimized doses of RO-318425 (10 µM), GF109203X (10 µM), Gö6976 (10 µM), PKCθ-IN (2.5 µM) or a mixture of Gö6976 (10 µM) and PKCθ-IN (2.5 µM). The cells were then stimulated for 10 minutes with agonists (Figure 2). For the posttreatment condition, the platelets were stimulated with indicated agonists. After 10 minutes, vehicle or indicated signaling inhibitors were added. Activation markers were measured 10 minutes later by flow cytometry. Shown are means ± SD (n ≥ 3) of raw data (PAC positive, % of control).

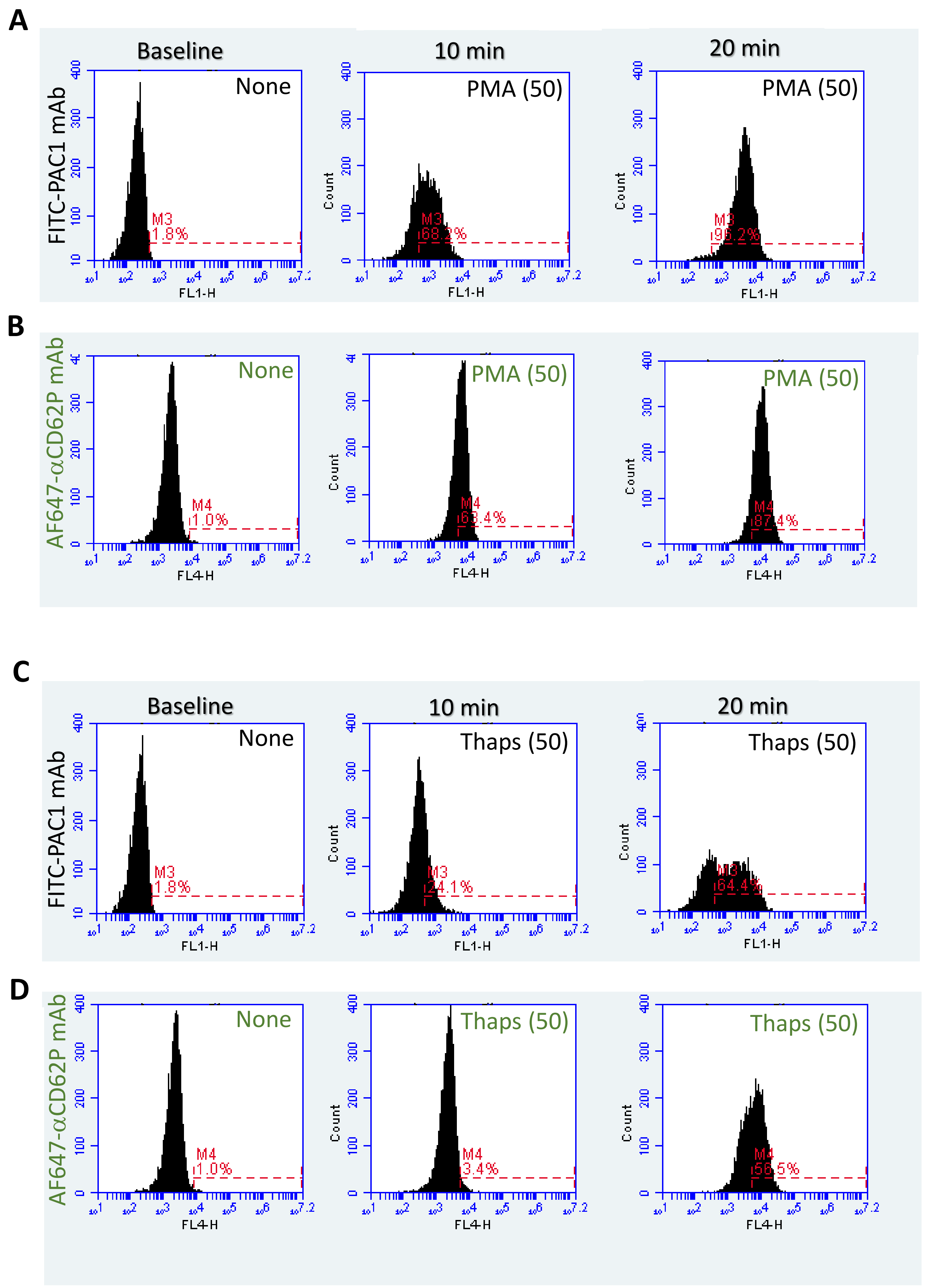

Suppl. Figure 3. Effects of PKC activation and elevated Ca^{2+} on integrin αIIbβ3 activation and P-selectin expression. Flow cytometry measurements of washed platelets, stimulated for 10 or 20 minutes with 50 nM PMA or 50 nM thapsigargin. Integrin αIIbβ3 activation (PMA **A,** thapsigargin **C**) and P-selectin expression (PMA **B**, thapsigargin **D**) were measured as for Suppl. Figure1.

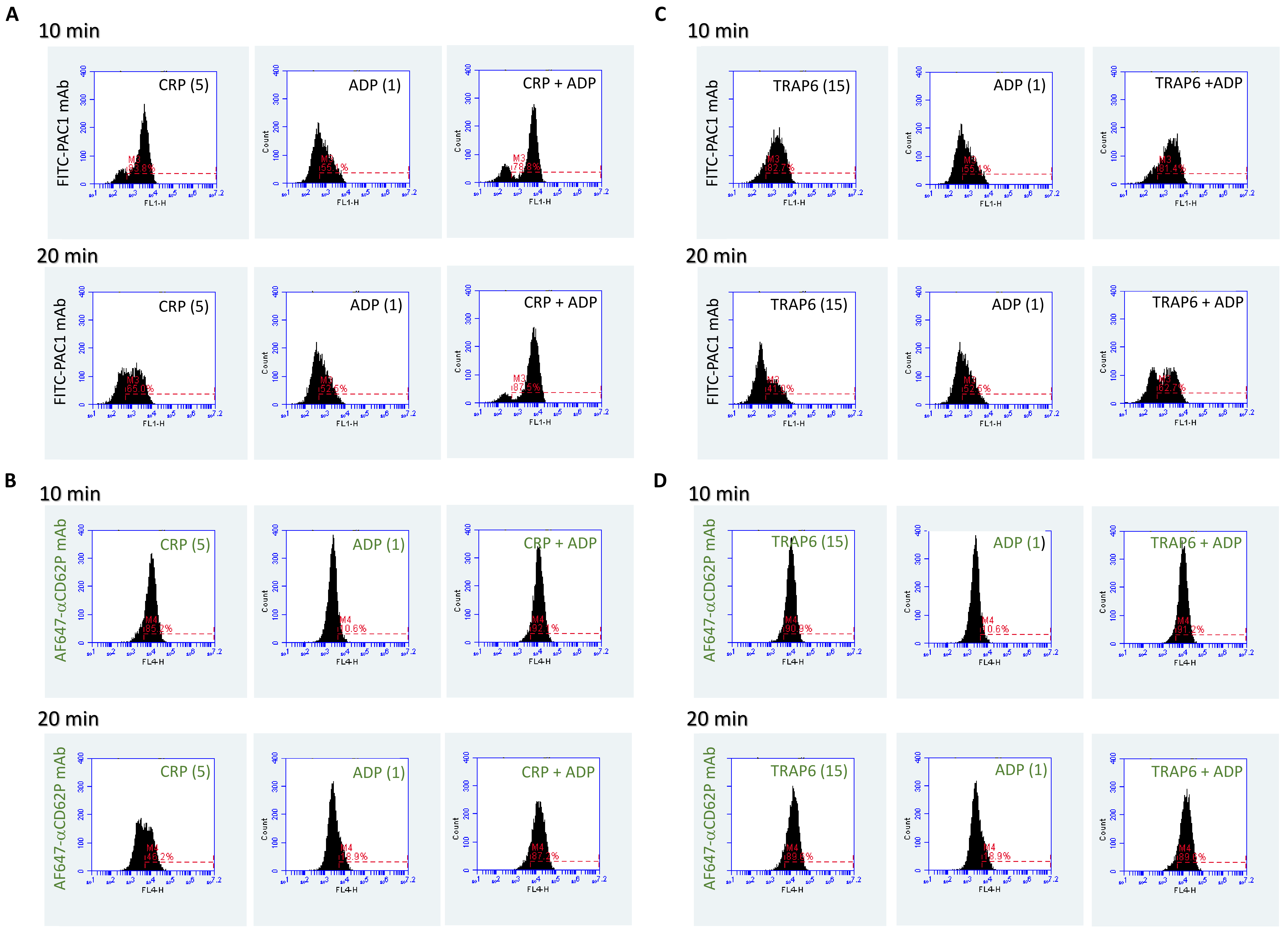

Suppl. Figure 4. Time-dependent effects of ADP receptor-dependent integrin αIIbβ3 activation and P-selectin expression. Platelets were stimulated for 10 or 20 minutes, as indicated, with CRP (5 µg/mL), TRAP6 (15 µM) and/or ADP (1 µM). Integrin αIIbβ3 activation and P-selectin expression were measured as for Suppl. Figure1, CRP and/or ADP (**A,B**), TRAP6 and/or ADP (**C,D**).

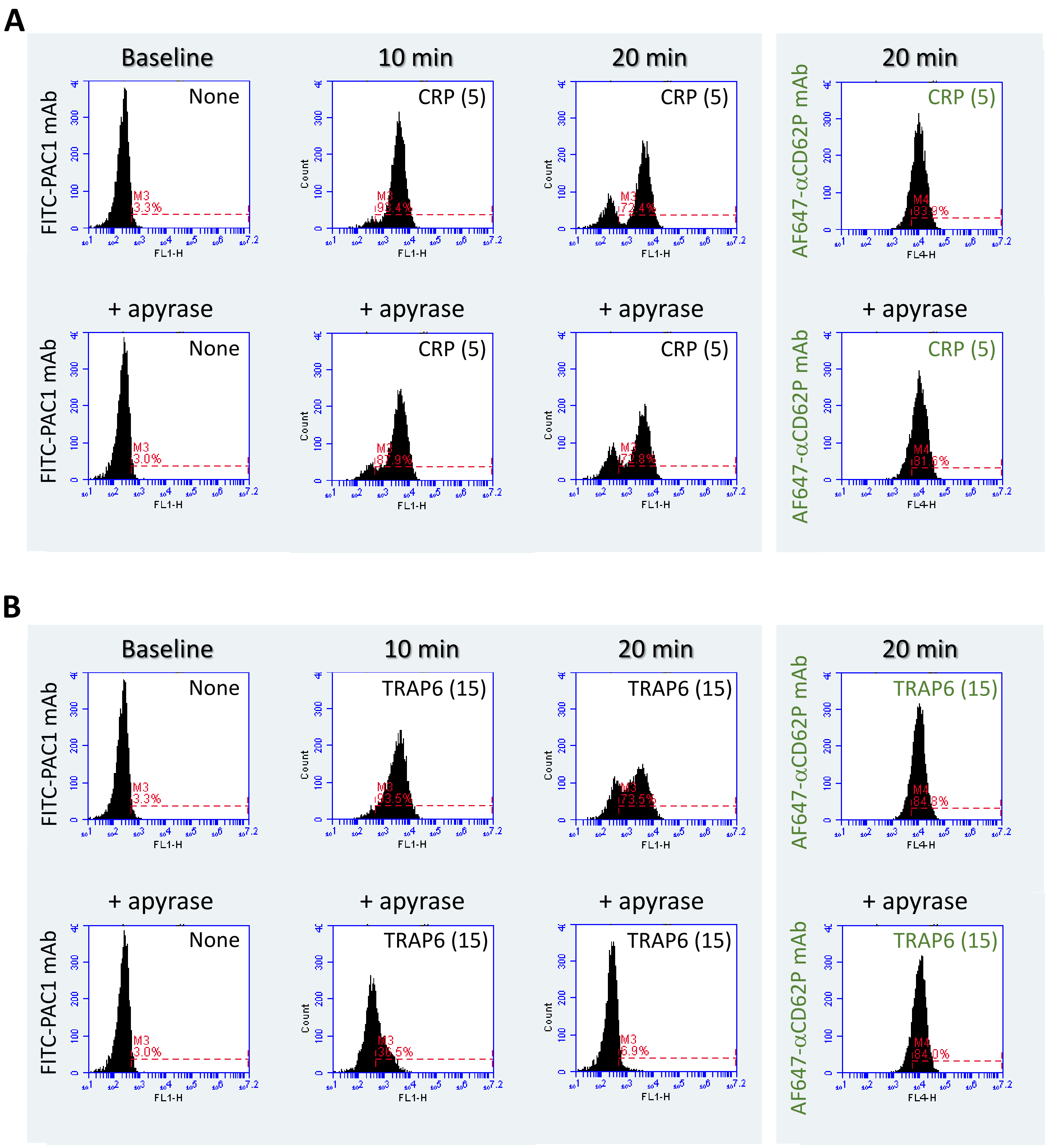

Suppl. Figure 5. Apyrase-affected integrin αIIbβ3 activation and P-selectin expression. Washed platelets were pre-incubated with ADP-degrading apyrase (1 U/mL) or DMSO, then stimulated for 10 or 20 minutes, as indicated, with CRP (5 μg/mL) or TRAP6 (15 μM). Integrin αIIbβ3 activation and P-selectin expression were measured as for Suppl. Figure1, CRP (**A**), or TRAP6 (**B**).

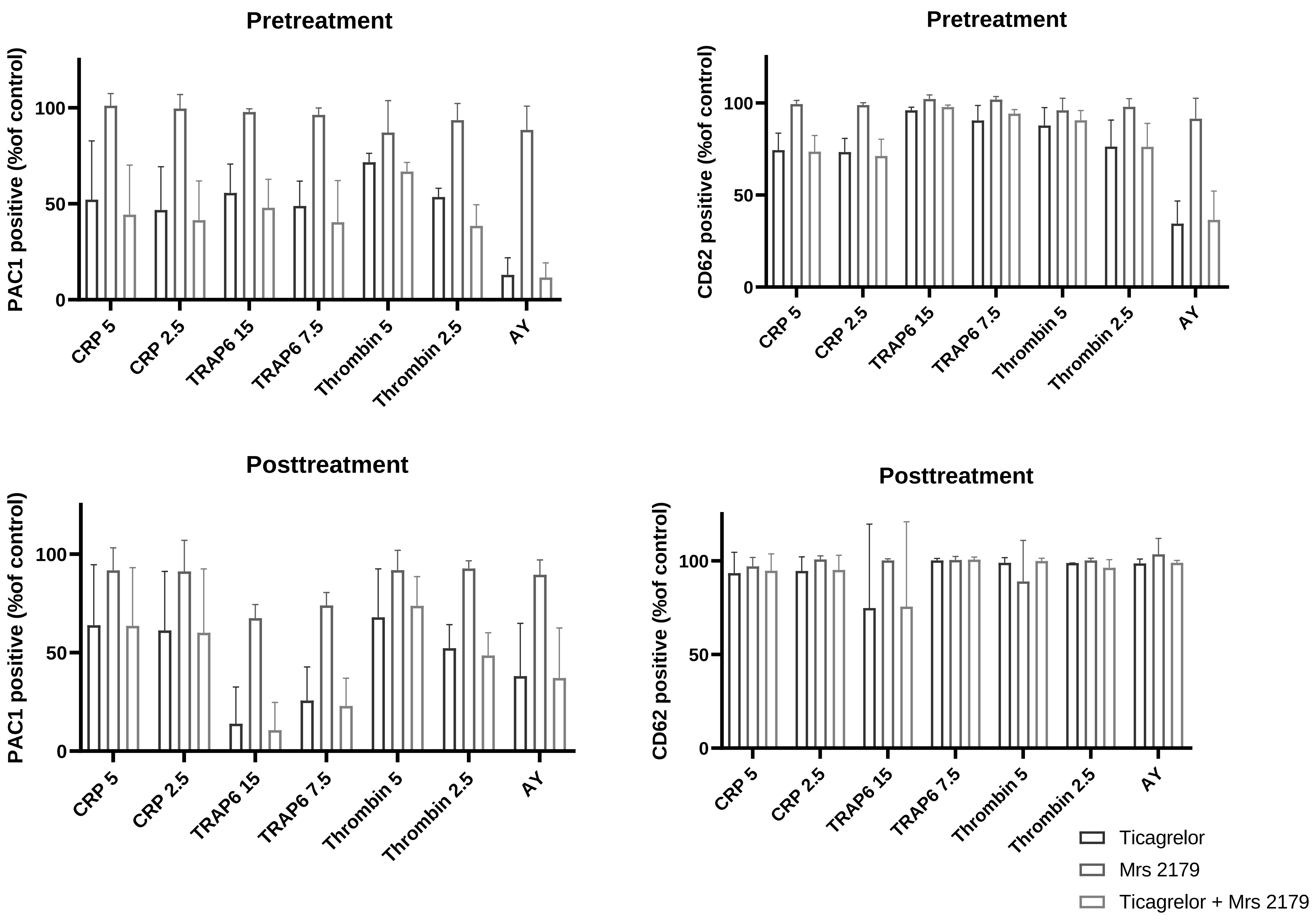

Suppl. Figure 6. Role of ADP on GPVI- and PAR-induced integrin αIIbβ3 activation and P-selectin expression. In the pretreatment condition, platelets were incubated for 10 minutes with vehicle (none) or optimized doses of ticagrelor (1 µM) and/or MRS-2179 (50 µM). The cells were subsequently stimulated with CRP (5 or 2.5 µg/mL), TRAP6 (15 or 7.5 µM), thrombin (5 or 2.5 nM) or AYPGKF (50 µM). In the posttreatment condition, the platelets were left unstimulated or stimulated with the same agonists. After 10 minutes, the platelets were posttreated with vehicle (none) or ticagrelor and/or MRS-2179. Flow cytometric detection was performed 10 minutes later, as in Suppl. Figure 1. Shown are means ± SD (n ≥ 3) of raw data (PAC positive, % of control).

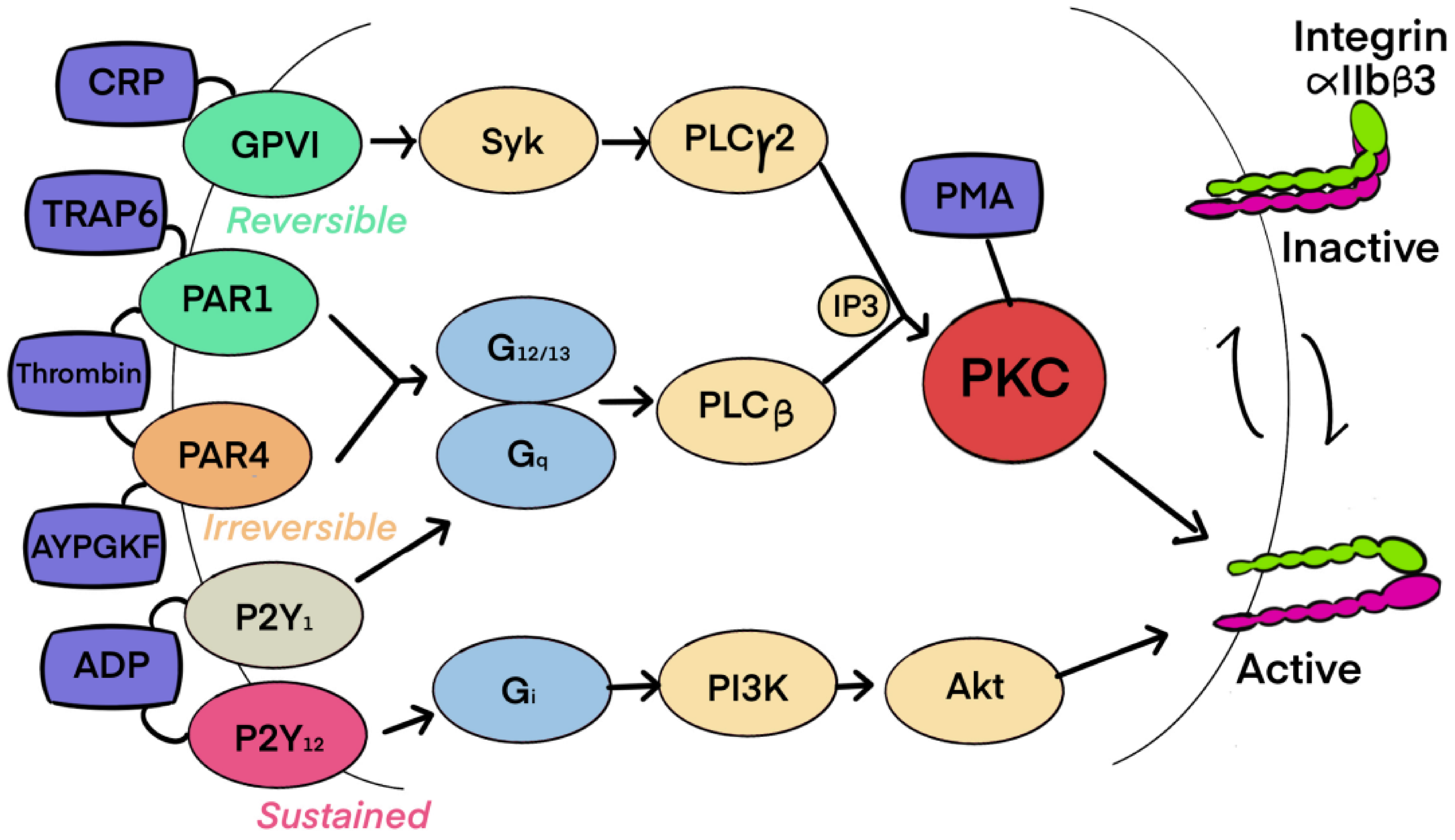

Suppl. Figure 7. Schematic presentation of investigated pathways involved in transient and persistent integrin αIIbβ3 activation. Highlighted are the reversibility of integrin activation upon GPVI and PAR1 signaling, while PAR4 stimulation results in persistent integrin activation. ADP and PKC signaling are critical for continued αIIbβ3 activity.
